# Marburg virus in Egyptian Rousettus bats in Guinea: Investigation of Marburg virus outbreak origin in 2021

**DOI:** 10.1371/journal.pntd.0011279

**Published:** 2023-04-26

**Authors:** Marat T. Makenov, Sanaba Boumbaly, Faya Raphael Tolno, Noumouny Sacko, Leno Tamba N’Fatoma, Oumar Mansare, Bonaventure Kolie, Olga A. Stukolova, Evgeny S. Morozkin, Ivan S. Kholodilov, Olga B. Zhurenkova, Marina V. Fyodorova, Vasily G. Akimkin, Anna Yu. Popova, Namoudou Conde, Mamadou Yero Boiro, Lyudmila S. Karan

**Affiliations:** 1 Department of Molecular Diagnostics and Epidemiology, Central Research Institute of Epidemiology, Moscow, Russia; 2 Virology Research Center/Laboratory of Viral Hemorrhagic Fevers, Conakry, Guinea; 3 International Center for Research of Tropical Infections in Guinea, N’Zerekore, Guinea; 4 Hospital of Guéckédou, Guéckédou, Guinea; 5 Department of Biology, Laboratory of Medical Zoology, University of Kindia, Kindia, Guinea; 6 Laboratory of Biology of Arboviruses, FSASI Chumakov Federal Scientific Center for Research and Development of Immune-and-Biological Products of RAS, Moscow, Russia; 7 Administration, Federal Service for Surveillance on Consumer Rights Protection and Human Wellbeing, Moscow, Russia; 8 Directorate Research Institute of Applied Biology of Guinea, Kindia, Guinea; Hokkaido University Research Center for Zoonosis Control, JAPAN

## Abstract

In 2021, a patient died from Marburg virus (MARV) disease in Guinea and it was the first confirmed case in West Africa. The origin of the outbreak has not been identified. It was revealed that the patient didn’t travel anywhere before the illness. Prior to outbreak, MARV had been found in bats in the neighboring Sierra Leone, but never in Guinea. Therefore, the origin of infection is unclear: was it an autochthonous case with spillover from a local population of bats or an imported case with spillover from fruit bats foraging/migrating from Sierra Leone? In this paper, we studied *Rousettus aegyptiacus* in Guinea as the possible source of MARV infection caused the patient death in 2021 in Guinea. We caught bats in 32 sites of Guéckédou prefecture, including seven caves and 25 locations of the flight path. A total of 501 fruit bats (Pteropodidae) were captured, including 66 *R*. *aegyptiacus*. The PCR screening showed three positive MARV *R*. *aegyptiacus*, roosting in two caves discovered in Guéckédou prefecture. After Sanger sequencing and phylogenetic analyses it was shown that found MARV belongs to the Angola-like lineage but it is not identical to the isolate obtained during the outbreak of 2021.

## Author summary

In 2020, MARV was first detected in fruit bats in Sierra Leone (West Africa). One year later, first outbreak of MARV in West Africa was registered in the neighboring Guinea after a man’s death in the Temessadou M’bokét village. The source of the outbreak has not been identified. It is known, that the reservoir animal of MARV is the cave-dwelling bat *Rousettus aegyptiacus*, and typically, MARV disease outbreaks emerge after a visit of the index patient to a cave or mine.

In our work, we studied bats in the vicinity of this village for the presence of the MARV. A cave hosting a colony of *R*. *aegyptiacus* was found close to the Temessadou M’bokét village (~4.5 km). Sampling and PCR screening of the bats from the colony identified two MARV-positive bats. The cave is inaccessible to humans; therefore, the index patient could not have been infected upon visit. We assume that the patient was infected outside the cave after direct or indirect contact with an infected bat of the same colony. These results provide the basis for preventive measures against new MARV outbreaks in Guinea.

## Introduction

In August 2021, in Temessadou M’bokét village (Guéckédou prefecture, Guinea), a patient was taken to a local health care point with symptoms of a hemorrhagic fever and died the next day. The case was investigated and attributed to Marburg virus disease (MVD) [1]. Sequencing of isolated MARV from the Guinean patient showed that outbreak was caused by the Angola-like Marburg virus (MARV) [1]. Previously, in 2017–2018, MARV was detected in Egyptian rousette bats (ERB; *Rousettus aegyptiacus*) in the neighboring Sierra Leone [2]. It was the first documented instance of MARV in West Africa. Phylogenetic analysis revealed that at least two genetic variants of MARV circulate in bats in Sierra Leone: Angola-like MARV and MARV genetically similar to variants from Uganda, Gabon, and the Democratic Republic of Congo (DRC) [2].

MARV belongs to the *Filoviridae* family and causes severe hemorrhagic infections in humans with a high mortality rate [3]. The Angola-like variant became known after the largest outbreak of MVD that occurred in 2005 in Angola, with 252 cases and 227 deaths [4]. The reservoir of MARV is ERB [5], that roost in caves forming large colonies [6,7]. MARV was also detected in the green monkey *Chlorocebus* sp. [8,9] and in the cave-dwelling insectivorous bats *Miniopterus inflatus* and *Rhinolophus eloquens* [10].

Thirteen MVD outbreaks had been described by 2019 [11,12]. All of them (with the exception of the two lab outbreaks in Germany and Russia) were of African origin. Epidemiological investigations of these outbreaks showed that seven MVD outbreaks (from 13) emerged after the index patient visited a cave, and the cave location was determined [11]. These include Sinoia cave in Zimbabwe, Kitum cave in Kenya, Python cave and Kitaka mine in Uganda [5,13–15]. Presence of large colonies of ERB has been confirmed in all of the caves. In case of some caves, MARV spillover events have occurred repeatedly. In particular, two outbreaks emerged after visiting Kitum cave in 1980 and 1987 (Kenya) [13,16], two separate spillover events occurred at Kitaka mine in 2007 (Uganda) [17] and two outbreaks were linked to Python cave in 2008 (Uganda) [14]. However, for some MVD outbreaks, including the largest outbreak in Angola in 2004–2005, the origin of human infection has not been identified [4].

In the Temessadou M’bokét case, it was not possible to know for sure whether the patient visited any caves or had been in contact with bats. ERB are capable of long-distance flights with maximum flying distance between 11–32 km, and documented maximum relocation distance of 500 km [18–20]. Just 56 km away from Temessadou M’bokét village is Koema Cave (Sierra Leone), where MARV has previously been found [2]. Therefore, theoretically, MARV could have been transferred from Sierra Leone to Guinea by infected ERB. On the other hand, it is possible that Guinea naturally has caves inhabited by MARV-positive ERBs and finding such caves is the most important task to prevent new MVD outbreaks.

In Guinea, the largest Ebola virus disease outbreak happened in 2013, which has sparked interest in finding animal reservoirs of the Ebola virus. Saéz et al [21] attempted to find the source of Ebola virus spillover [21]. The authors began their study in the village of Meliandou in Guéckédou prefecture, where the index patient lived and died. They caught and screened 169 bats of 13 species and obtained negative results [21]. In addition, screening of bats for filoviruses was carried out in Guinea [22] after the discovery of Bombali virus (*Bombali ebolavirus*) in the neighboring Sierra Leone [23]. MVD outbreak in Guinea in 2021 reinvigorated the search for filoviruses in this country, and this time both *Ebolavirus* and *Marburgvirus* genera were involved in the search. A team of WHO specialists arrived in Temessadou M’bokét in August-September 2021 as part of epidemiological investigation of the case in search of the MARV reservoir [24]. The team trapped the bats in the vicinity of the village of Temessadou M’bokét, as well as in the vicinity of neighboring villages of Baladou Pebal and Koundou [24]. However, no public information about the detection of MARV-positive bats in Guinea has been released.

In this paper, we studied *R*. *aegyptiacus* in Guéckédou prefecture and found the most likely source of infection of the patient who died there from MVD in 2021.

## Methods

### Ethics statement

The procedures used in this study adhered to the tenets of the Declaration of Helsinki. Approval was obtained from the National ethics committee for Health Research of Guinea (document number 061/CNERS/15), and from Research Institute of Applied Biology of Guinea (document number 14/DG/IPG/K/2019).

### Bat capture and processing

The bats were captured in June-July 2022. Two-step strategy was utilized to search for the ERB roosting caves. First, bats were captured on flight paths using mist nets. Second, bats were caught by setting nets at the entrance and in close proximity to the caves. The search for the caves was carried out based on bat captures on flight paths. In the event of ERBs presence in the captures, local residents from nearby villages were questioned if there were caves in the vicinity.

Bat species were identified based on morphology [25]. Species identification of ERB was confirmed genetically, by sequencing of the cytochrome-b (cyt-b) fragment of voucher specimen (GenBank accession number OQ383610) using the primer pair LGL 765F and LGL 766R [26]. Following Amman et al [12], we divided all caught ERB into two age groups: juveniles (under 6 months of age) with forearm lengths less than 90 mm and adults with forearm lengths greater than 89 mm. Standard safety guidelines were used for handling and sampling of mammals that are potentially infected with infectious pathogens [27]. Captured bats were placed in breathable cotton bags, which were moisturized to prevent animal dehydration, and transported to the field laboratory where bats were euthanized using standard protocols. Blood from the euthanized bats was collected through cardiac puncture into sterile tubes with 0.5 M EDTA. Blood samples were centrifuged to separate plasma and cells. Urine was collected antemortem in sterile Petri dishes or, postmortem, with a sterile syringe from the bladder. During necropsy, samples of skin, sections of the brain, lymph nodes, salivary glands, liver, spleen, kidney, lung, and intestines were obtained and stored in RNA tissue protect buffer (RNAlater, Qiagen, Hilden, Germany) at– 20°C. Before dissection, all animals were examined for the presence of arthropod ectoparasites. All detected ectoparasites were placed in 70% ethanol and stored at– 20°C. Collected bat flies were identified morphologically [28] and by sequencing of the cytochrome oxidase subunit 1 gene (GenBank accession numbers OQ196106-OQ196108) using primers ST-COI-F2 and jgHCO2198 [29,30]. Pools of internal organs (liver, spleen) and lymph nodes of bats were homogenized with TissueLyser LT (Qiagen, Hilden, Germany) in 0.5 ml of 0.15 M NaCl solution immediately after necropsy. Total RNA was extracted from 50 μl of 10% suspension using an RNeasy Plus kit (Qiagen, Hilden, Germany).

### Viral detection and sequencing

Detection of filovirus RNA was performed using an Amplisens FiloA-Fl commercial kit (Central Research Institute of Epidemiology, Moscow, Russia) designed for the detection of *Zaire ebolavirus*, *Sudan ebolavirus*, and Marburg virus. For sequencing, total RNA from skin homogenates, ectoparasites, oral swabs, blood plasma, urine, and unpooled organ tissues (brain, liver, spleen, lung, kidney, lymph nodes, and salivary glands) for PCR-positive animals was re-extracted using the same preparation and RNA extraction method. Extracted RNA was studied using the same qRT-PCR kit and positive samples were reverse transcribed using a Reverta-L RT kit (Central Research Institute of Epidemiology, Moscow, Russia) according to the manufacturer’s instructions.

Four gene fragments of MARV were Sanger sequenced using the primers shown in Table 1. One qRT-PCR-positive sample with a low RNA concentration was sequenced only with the primers used in the qRT-PCR screening. Purified PCR products were sequenced bidirectionally using a BigDye Terminator v1.1 Cycle Sequencing kit (Thermo Fisher Scientific, Austin, TX, USA) on Applied Biosystems 3500xL Genetic Analyzer (Applied Biosystems, Foster City, CA, USA). Nucleotide sequences were aligned with CLUSTALW algorithm in MEGA X [31]. Phylogenetic analyses were performed using maximum-likelihood method with the best-fit substitution model (T93+G). Support for the tree was assessed with 1000 bootstrap replicates. Consensus trees were reconstructed with MEGA X and visualized in iTOL v6.6 (https://itol.embl.de).

**Table 1:** Primers used for sequencing of qRT-PCR positive samples.

Primer	Nucleotide sequence (5’–3’)	Target	Reference
MBG704F1	GTAAAYTTGGTGACAGGTCATG	NP gene	[2]
MBG1248R1	TCT CGTTTCTGGCTGAGG		
MBG719F2	GGTCATGATGCCTATGACA GTATCAT		
MBG1230R2	ACGGCIAGTGTCTGACTGTGTG		
MARV-F4	TCTGCAGACAAATCCTCCAGAATCACTT	NP gene	Self-designed
MARV-R4	TCAGCATATTCTCTGAATCAAGTCAT		
MBGVP35-F1	GCTTACTTAAATGAGCATGG	VP35 gene	[2]
MBGVP35-R1	AGIGCCCGIGTTTCACC		
MBGVP35-F3	CAAATCTTTCAGCTAA GG		
MBGVP35-R2	TCAGATGAATAIACACAIACCCA		
MARV-F13	TCCACGTGCAAGATCTATGAGC	VP30 gene	Self-designed
MARV-R13	CAGGCAAGTTGTAACGCGTTGAT		
MARV-F17	ACCACACATGATTTGCATTTGAC	L-gene	Self-designed
MARV-R17	TCACCCATGACACTAGACTT		

## Results

### Searching for *Rousettus aegyptiacus* roost caves

During this study, we caught bats at 32 sites in Guéckédou prefecture, including seven caves and 25 locations of the flight path. A total of 501 fruit bats (Pteropodidae) were captured, including *Lissonycteris angolensis* (224 bats), *Myonycteris leptodon* (110 bats), *R*. *aegyptiacus* (66 bats), *Epomops buettikoferi* (82 bats), *Hypsignathus monstrosus* (13 bats), *Eidolon helvum* (4 bats), and *Micropteropus pusillus* (2 bats). Detailed data on the caught animals can be found in S1 Table. We started our investigation from the Temessadou M’bokét cave, which is closest to the Temessadou M’bokét village.

#### Temessadou M’bokét cave

(N 8.61706°, W 10.36345°) is located 600 m away from Temessadou M’bokét village, where MARV-positive patient died in 2021. It is a shallow talus cave that was formed between large boulders. Examination of the cave and captures at its entrances showed that it was inhabited by *L*. *angolensis*, *Hipposideros caffer/ruber*, and *Hipposideros beatus*, but no ERB were found in the cave. It is known that *R*. *aegyptiacus* prefer deep and dark caves for roosting in the daytime and do not inhabit talus caves [6]. Thus, we assume that ERB were not simply too rare or absent from the area at the time of the study, but in general ERB would not find it attractive for roosting. Therefore, it is unlikely that the MARV-positive patient became infected while visiting this cave.

After investigation of the first cave, we used a two-step algorithm in which the first bats were trapped in flight paths, and after capturing the ERB, the surroundings were investigated for the presence of caves. Using this strategy, ERB were trapped in flight paths in eight different locations, and at the time of the study two caves with ERB roosts were discovered.

#### Legon Tyo cave

**Fig 1:**
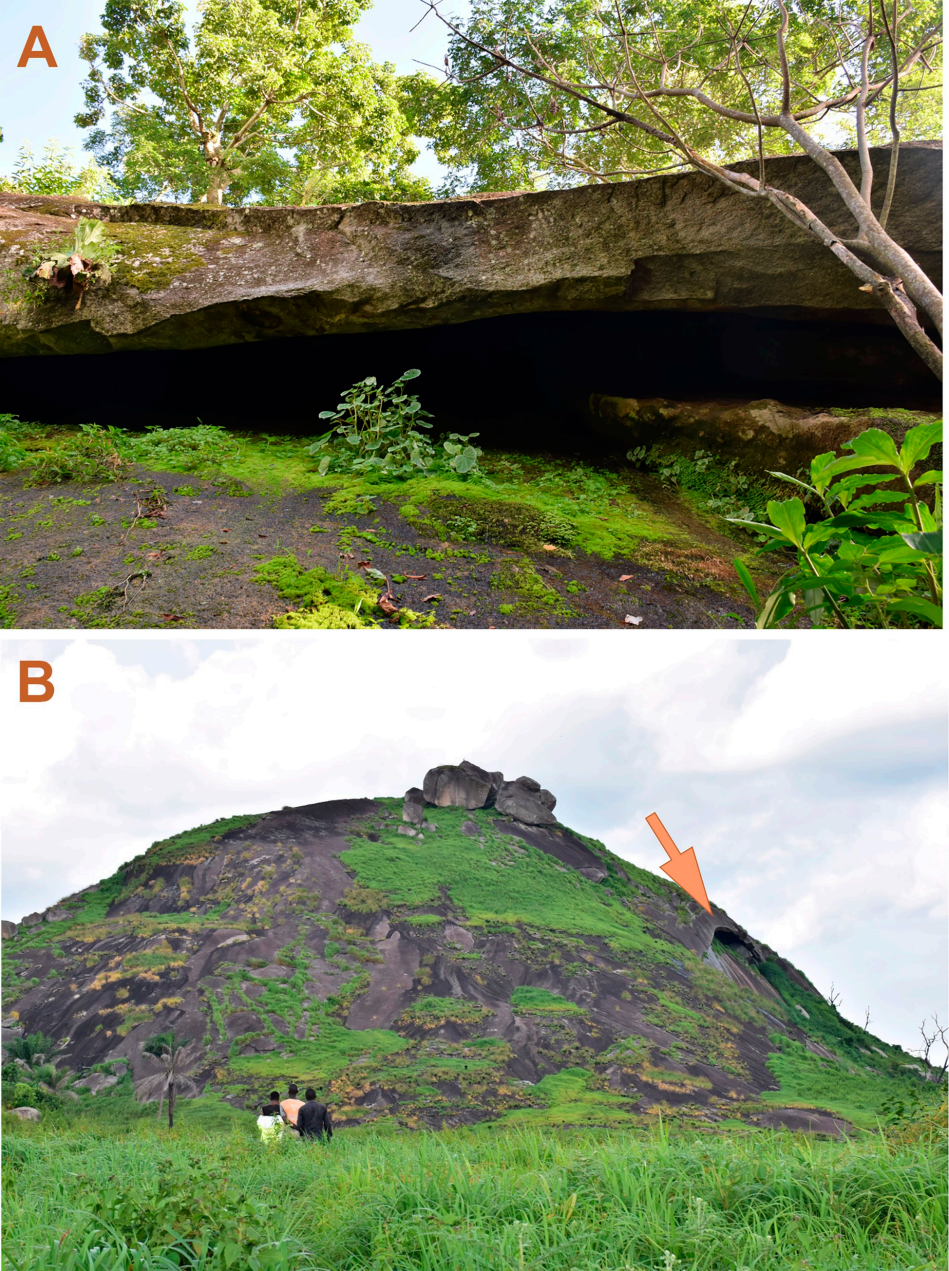
The Legon Tyo cave. Panel A: The entrance to the Legon Tyo cave (view from below). The cave is located on a wooded slope surrounded with tall trees that block the view and prevent taking a general view photo. Therefore, the picture of the cave entrance was taken at close range. The entrance to the cave hangs from above with a cornice (The growth direction of the tree in the right corner of the photo helps to understand the orientation of the photo). **Panel B:** General view of a similar cave located on the opposite (southern) slope of Mount Legon Tyo. This cave has a similar entrance structure, but unlike the Legon Tyo cave, here the slope is not covered with trees and therefore it is clearly visible that the entrance is located high on a steep slope and a person cannot get inside without special equipment.

(N 8.64561°, W 10.37876°) is located in Koundou subprefecture, close to Tongolo village (~600 m), on the northern slope of Legon Tyo Mountain. Near the entrance to the cave, the mountainside is covered with small plantations of rice, pepper, cassava and other plants cultivated by locals. There are several talus caves on the slope in front of the entrance to the main cave. *M*. *leptodon* and *L*. *angolensis* roosted in these small cavities between large boulders. The entrance to the main cave is located 50 meters above the talus caves up on an almost sheer slope and is not accessible to humans (Fig 1). We caught 19 ERB near the cave entrance, as well as on the flight paths in the vicinity of the cave (Table 2). Temessadou M’bokét village, where the 2021 MVD outbreak emerged, is located 4.5 km from this cave. An important detail is that the cave is located on the lands of the farmers of Tongolo village, and the movement of outsiders (including residents of neighboring villages) through the area is limited. The farmer who works the land near the cave has denied that it had been visited by any outsider.

**Table 2:** *Rousettus aegyptiacus* trapping data with the results of PCR screening on MARV.

Roosting cave	Number of *R*. *aegyptiacus* / Number of MARV+		
	Captured in the entrance of the cave	Captured on flight paths	Total
**Legon Tyo cave-related animals**	**13 / 1**	**6 / 1**	**19 / 2**
Juveniles	4 / 1	2 / 0	6 / 1
Adults	9 / 0	4 / 1	13 / 1
**Kokongoa cave-related animals**	**14 / 1**	**26 / 0**	**40 / 1**
Juveniles	1 / 1	8 / 0	9 / 1
Adults	13 / 0	18 / 0	31 / 0
**Animals for which the roosting cave was not found**	**0 / 0**	**7 / 0**	**7 / 0**
Juveniles	0 / 0	4 / 0	4 / 0
Adults	0 / 0	3 / 0	3 / 0
Total	27 / 2	39 / 1	66 / 3

#### Kokongoa cave

(N 8.64561°, W 10.37876°) is located 48 km away from Temessadou M’bokét village on the northeastern slope of Mount Kokongoa (Tekoulo subprefecture). The cave is structured as a wide tunnel with two large entrances located at elevations of 642 and 605 meters above sea level, respectively. People from the surrounding villages visit this cave for ritual sacrifices at least once a year (usually in January) and store their ceremonial material directly at the lower entrance of the cave. Visual inspection of the cave, as well as trapping results with mist nets installed at both entrances, confirmed the presence of a colony of ERB in the cave, alongside other bats species such as *Hipposideros ruber/caffer*, *Hipposideros beatus*, and *Hipposideros jonesi*. A total of 40 *R*. *aegyptiacus* were caught at the cave entrance and in the surrounding area (Table 2).

**Fig 2:**
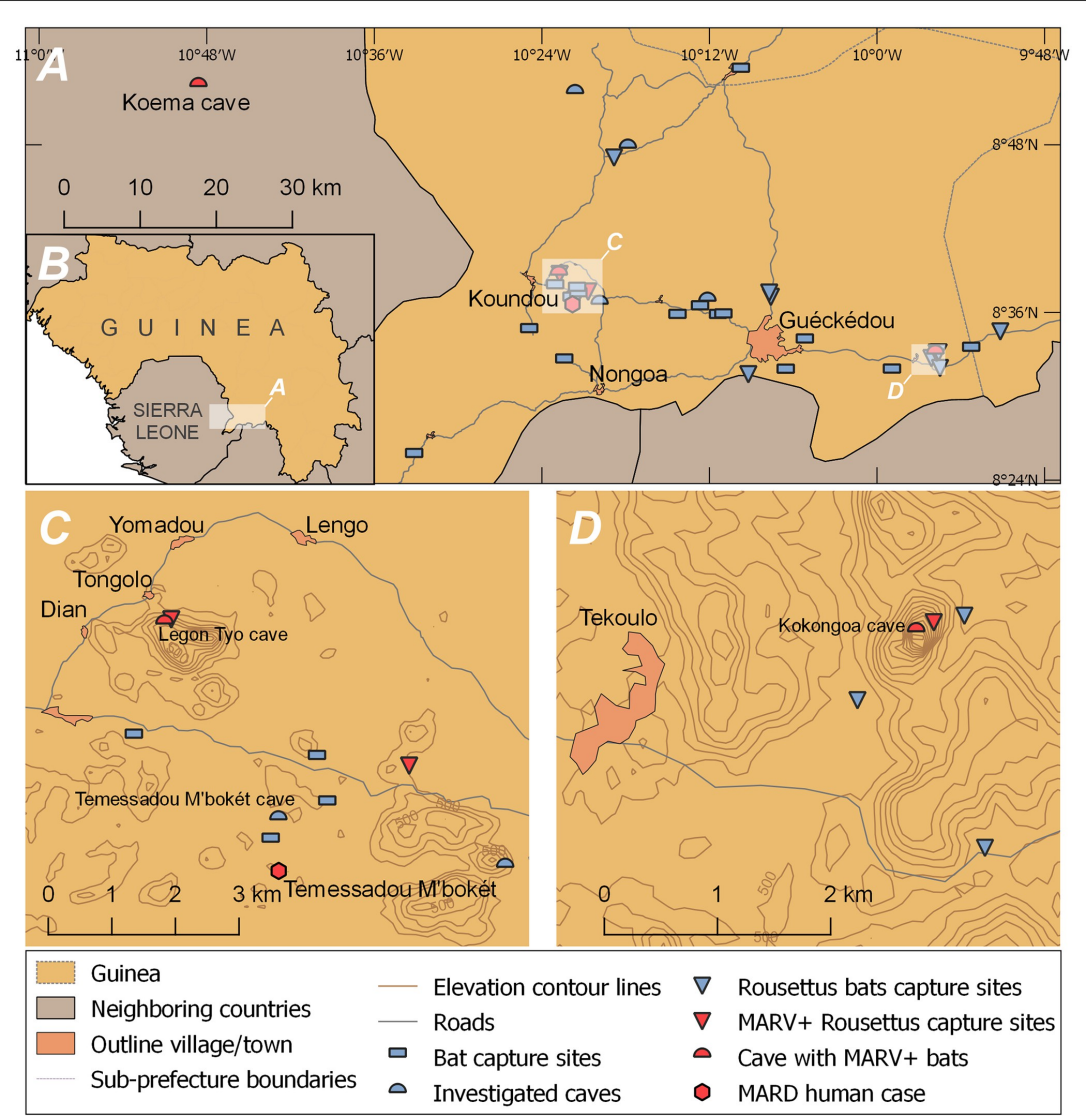
Sampling locations depicting the caves where PCR-positive MARV bats were caught. Map A shows the sampling locations in Guéckédou prefecture (Guinea) indicating the Koema cave in Sierra Leone where MARV-positive bats were previously found [2]. Map B shows an overview of the sampling area and Guinea. Map C focuses on the area around Legon Tyo Cave and Temessadou M’bokét village. Map D zooms on the area around the Kokongoa cave. Elevation contours on maps C and D are shown for altitudes over 440 m with an interval of 20 m. The base vector layer of the map was downloaded from Natural Earth (https://www.naturalearthdata.com/downloads/10m-physical-vectors/).

For seven ERB caught on flight paths in three different locations (Fig 2) no roosting cave could be found. Additionally, we caught bats in 11 locations within a 20 km radius of Temessadou M’bokét village, including three caves (Fig 2), but other ERB colonies were not found in this area in either the caves or the flight paths.

### PCR detection of MARV in *Rousettus aegyptiacus* and Sanger sequencing

During the screening stage, homogenates of lymph nodes and pools of sections of internal organs from all trapped ERB were tested for the presence of MARV RNA. Only three samples were shown to be positive: two from bats were trapped at the entrance or in the vicinity of the Legon Tyo cave, and one from a bat captured at the entrance of the Kokongoa cave (Table 2). The lowest Ct value was present in a juvenile female ERB from Legon Tyo cave (bat id: ERB-452), particularly in the spleen tissue and axillary lymph nodes (Ct values 27.1 and 28.1, respectively) (Table 3). We collected four bat flies, *Eucampsipoda africana*, from this animal and tested them separately. All four bat flies were PCR-negative for MARV. For the other two MARV-positive ERBs, the Ct values were both approximately 35. Oral swabs of all three PCR-positive ERB gave negative MARV results.

**Table 3:** Detailed data on MARV-positive *Rousettus aegyptiacus*.

Location	Bat ID	Sex	Age status	RT–PCR Ct value	Sequencing data
Legon Tyo	ERB-79	Male	Adult	Lymph node– 35.0 Lung, kidney, liver, spleen–negative	GenBank accession number: OP729425
Legon Tyo	ERB-452	Female	Juvenile	Spleen– 27.1 Liver– 32.2 Kidney– 32.1 Lymph node, axillary– 28.1 Lymph node, mesenteric– 31.8 Salivary glands– 30.3 Ectoparasites*, oral swab, brain, lung–negative	GenBank accession numbers: OP716850-OP716853
Kokongoa cave	ERB-387	Female	Juvenile	Liver+spleen– 35.2 Lung, kidney, lymph node–negative	No

*–four bat flies (*Eucampsipoda africana*) were collected and tested

**Fig 3:**
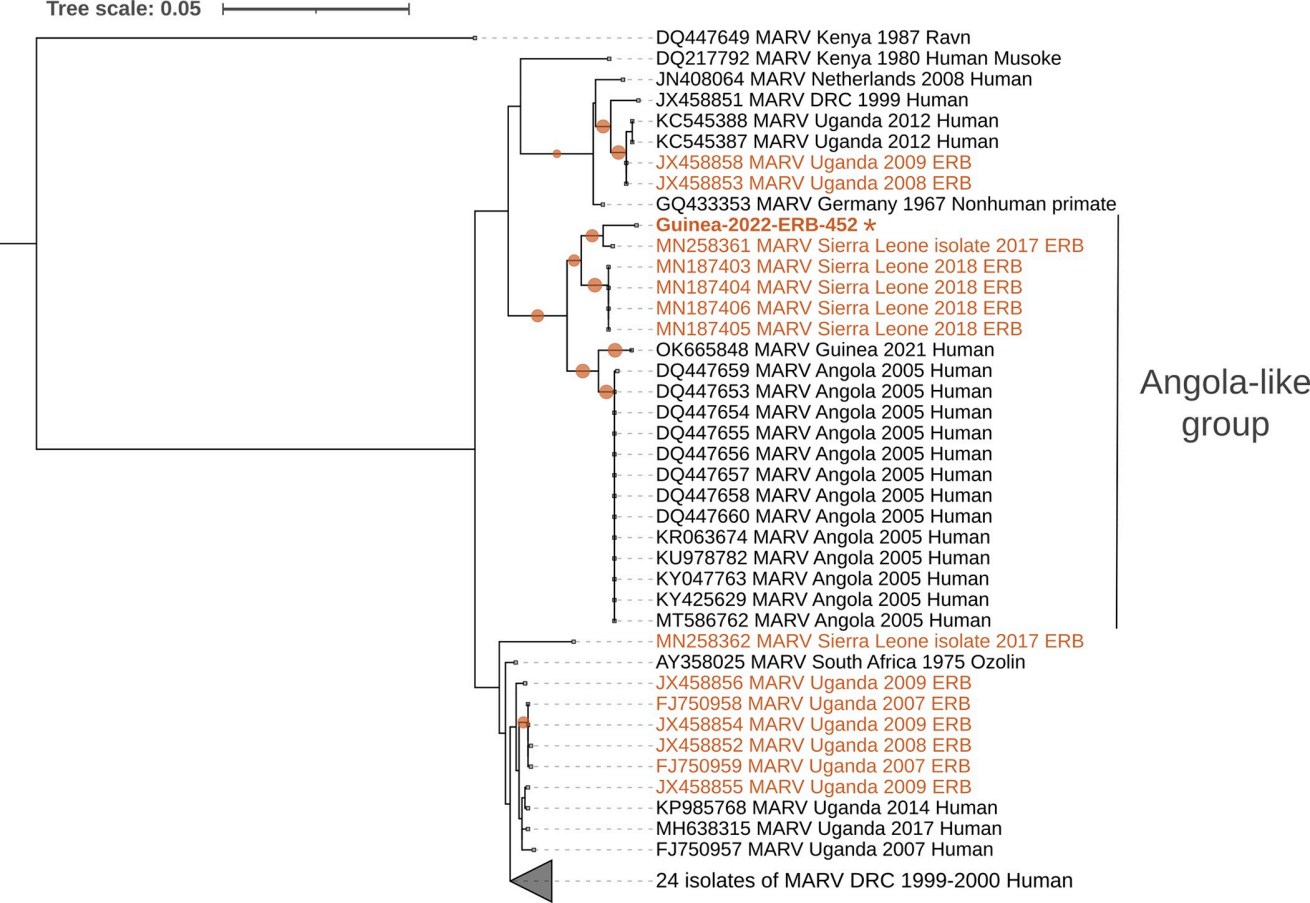
Maximum-likelihood phylogeny of 64 partial Marburg virus polymerase gene (L gene) fragments with a total length of 1262 bp. Tree was constructed using the best-fit nucleotide substitution model (T93+G). Sequences in brown were obtained from ERB, and sequences in black come from human and nonhuman primate samples. The sequence from *R*. *aegyptiacus* ID ERB-452 generated in this study is indicated with an asterisk and bold text. Scale bar indicates the mean number of nucleotide substitutions per site. The filled circles on branches indicate bootstrap values greater than 0.9.

We obtained sequences for fragments of the nucleoprotein (NP), viral protein 35 (VP35), viral protein 30 (VP30), and polymerase (L) genes from the RNA isolated from the ERB-452 bat with (Legon Tyo cave). Phylogenetic analysis of the polymerase gene (1262 bp) fragments showed that the identified isolate is most similar (p-distance = 0.012) to the sequence of MARV found in ERB from Sierra Leone (GenBank accession number MN258361, Fig 3) and clustered with the Angola-like group. Phylogenetic analysis for other fragments of viral genome supported these results: sequences of NP gene (two fragments with 459 bp and 457 bp of length), VP35 (300 bp), VP30 (1107 bp) also clustered with the Angola-like group (S1 Appendix).

**Fig 4:**
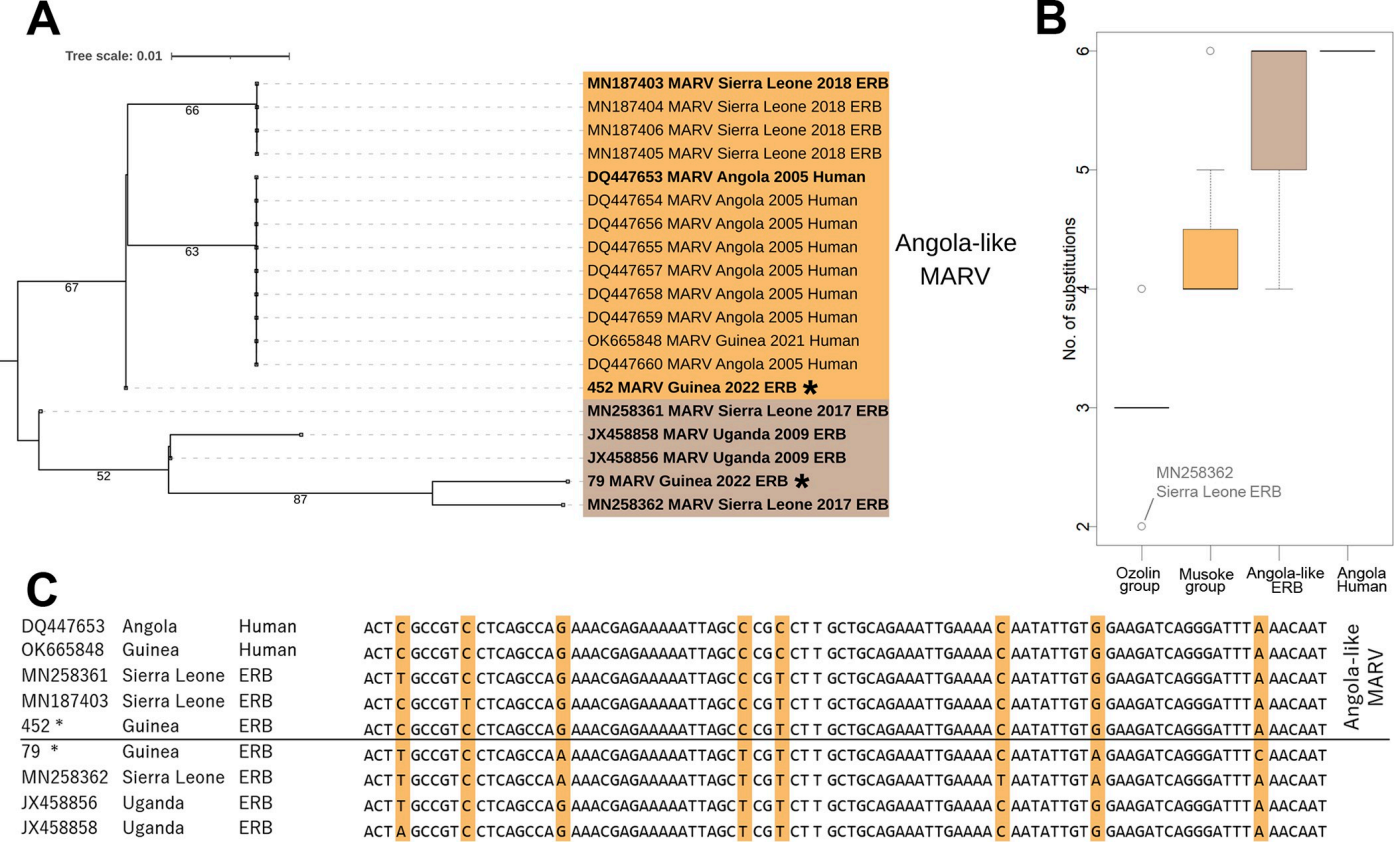
Phylogenetic tree, distances, and alignment comparing the relationship of the MARV sequence of sample ERB-79 to other marburgviruses. **The sequences from Guinea generated during this study are indicated with asterisks. A:** Maximum-likelihood tree (Tamura 3-parameter model with 1000 pseudoreplicates) of partial marburgvirus nucleoprotein gene fragments (92 bp). Scale bar indicates the mean number of nucleotide substitutions per site. The sequences in bold were used in the alignment presented below. The tree is midpoint rooted and the root position is verified by the Ravn virus (DQ447649) outgroup. **B:** Phylogenetic distances between sample ERB-79 and MARV sequences of different phylogenetic groups that were available in GenBank (35 sequences of Ozolin group, 8 sequences of Musoke group, 6 sequences of Angola-like ERB group, and 13 sequences of Angola human group). Distance is shown as the number of substitutions (on the 92 bp fragment of the NP gene). Solid line represents the median, box shows interquartile range (IQR), whiskers represent 1.5 × IQR, and the circles indicate outliers (values exceeded 1.5 × IQR). **C:** Aligned fragments (92 bp) of the MARV nucleoprotein gene with highlighted variable sites.

For the bat ERB-79 (Legon Tyo cave), only 92 bp amplicon obtained using screening qRT-PCR was sequenced (Fig 4, panel C) as longer fragments could not be generated. Analysis of this short fragment using BLAST showed that it belongs to the Marburg virus nucleoprotein in the exact region flanked by the primers of the qRT-PCR kit, with no matches to any other sequences in GenBank. Thus, we concluded that the obtained PCR product was specific, and that sample from ERB-79 did indeed contain MARV RNA.

Although samples ERB-79 and ERB-452 were obtained from animals that belong to the same colony, their MARV sequences were not identical. Sequence on the 92 bp fragment of NP gene from ERB-79 has five single-nucleotide substitutions that distinguish it from ERB-452 (Fig 4, panel C). Four are synonymous, but the substitution at position 1326 (according to the sequence AY358025 Marburg virus strain M/S.Africa/Johannesburg/1975/Ozolin) is nonsynonymous and leads to the replacement of lysine by glutamine. This substitution is unique and separates the ERB-79 sequence from the other MARV sequences available from GenBank. The most closely related sequence harboring only two substitutions was the virus previously found in ERB in Sierra Leone (GenBank accession number MN258362) (Fig 4, panel C). The full-length sequence of this isolate was clustered outside of Angola-like group and is close related to MARV from Uganda and DRC [2]. For convenience, we refer to this group as Ozolin group, after the strain M/S.Africa/Johannesburg/1975/Ozolin. The sequence from ERB-79 (92 bp) is insufficient to plot a phylogenetic tree with a robust topology (Fig 4, panel A). However, comparison of the genetic distances between ERB-79 and other MARV sequences available in GenBank shows its similarity to the isolates of the Ozolin group (mean 3.0 substitutions, p-distance = 0.033, N = 35), and distance from the isolates of the Angolan outbreak 2005 and Angola-like sequences from ERB (mean 6.0 and 5.5 substitutions, respectively) (Fig 4 panel B). Thus, we cannot confidently conclude that ERB-79 sample belongs to a specific MARV lineage, but we can state that at least this short fragment of its genome bears substitutions that are not typical for Angola-like MARV.

We were unable to obtain sequences from specimen 387 (Kokongoa cave). We assume that sample ERB-387 initially had a low viral load, and after freeze/thaw and re-extraction, viral RNA has decreased below the detection threshold of the PCR kit.

## Discussion

In this work, we confirm the presence of MARV in ERB in Guinea. Moreover, we found MARV-positive ERB in close proximity (4.5 km) to the Temessadou M’bokét village, where an MVD outbreak emerged in 2021. However, this patient could not have been infected while visiting the roosting cave of these ERB, as it is inaccessible to humans. Thus, we can suggest the two most likely MARV spillover scenarios in the 2021 outbreak. First is a direct contact with the infected ERB outside the cave during hunting, catching, or finding a weakened animal along the bat flight paths and/or near its feeding grounds. This assumption is supported by the work of Saéz et al. [21], which showed that local people of Guéckédou prefecture eat bats, hunting them with guns, sticks or machetes and even catching them by hand. The second possibility is an indirect spillover by eating infected fruits or through contact with an infected primate that fed on contaminated fruits. ERB do not typically eat the entire fruit; rather, they chew it to extract the juice and then discard the pulp [32,33]. Additionally, they may urinate on a fruit or drop the fruit after test bite [6,32]. This feeding behavior, combined with the ability of infected ERB to shed the virus in urine and saliva [34–37], means that MARV can be spread through fruits that ERB have previously eaten. Amman et al. [32] confirmed the feasibility of this transmission way by showing that MARV is stable on fruits for at least six hours. Based on the above, we can consider that this spillover scenario for the 2021 MVD outbreak is also very likely.

It is possible that there was another ERB colony in the vicinity of Temessadou M’bokét village that roosted in a cave that we could not find. However, our survey of local residents showed that they are not aware of other caves in the area. Until other caves are discovered, the ERB colony in Legon Tyo cave remains the closest documented source of MARV around the Temessadou M’bokét village (4.5 km). Assuming that the patient from the village got the virus from the ERB of the Legon Tyo colony, then the 2021 MVD outbreak emerged after contact with the virus outside the cave.

Detection of MARV-positive fruit bats in the Kokongoa cave, far from the Legon Tyo cave (50 km) and Temessadou M’bokét village (48 km), indicates that MARV is probably more widespread and more caves with infected ERBs exist in Guinea. Perhaps other outbreaks of MVD have previously occured in Guinea before but they have gone unnoticed or undocumented. Therefore, we consider that it is important to investigate and monitor ERB colonies in other prefectures of Guinea (Nzérékoré, Beila, Macenta, Youmu, Lola, Madina Oula, Mamou, Kindia, etc.). Moreover, similar research should also be carried out in other countries of West Africa (Liberia, Côte d’Ivoire, Mali, Burkina Faso, etc.). The importance of this action is highlighted by the Ghana MVD outbreak in 2022 [38], which showed that MARV is more widespread in West Africa than previously known.

We caught three MARV-positive ERB, and two of them were juveniles. Due to the low number of samples, we could not explore any age bias. However, Amman et al. [2] previously demonstrated significant age bias among MARV-positive bats in neighboring Sierra Leone: all 11 PCR-positive bats were juveniles. Furthermore, it was confirmed previously that juvenile ERB are more frequently infected by MARV than adult animals [12,14]. Therefore, we can expect a similar age bias in Guinea, with a predominance of juveniles among MARV-positive ERB. Thus, to prevent possible new outbreaks of MVD, it is important to not only know the location of the caves but also the seasonality of ERB breeding [12]. In sub-Saharan Africa, the ERB has biannual birthing seasons [6,39]. We assume that in Guinea, as well as Uganda, the reproductive chronology of ERB is a seasonal bimodal polyestry without postpartum estrus [40,41]. Females have two litters per year and a gestation of 106 days [6]. Females are in reproductive synchrony; therefore, young are born in two distinct seasons of parturition. Based on observations in Liberia [42–44] and Sierra Leone [2], fruit bats in Guinea have two parturition seasons, with peaks in December and June. After three months, the puppies fly out of the cave on their own for the first time and gradually begin to explore the area around the cave [45]. At this time, juveniles pose the greatest danger, as they significantly increase the likelihood of a spillover event. Amman et al. [12] showed that juvenile ERB of approximately 4.5–7.5 months of age are most likely to be infected with MARV. According to our assumptions, in Guinea these high risk periods fall on April-July and October-January. This is supported by with the timing of the 2021 outbreak: the first MVD symptoms in the patient appeared on July 25 [1]. This is also consistent with the data from Sierra Leone, where MARV-positive juveniles were caught in September-October and December [2].

Phylogenetic analysis of sequences obtained in this study revealed two main outcomes. The first result is the detection of an Angola-like MARV’s RNA in an ERB caught 4.5 km from the Temessadou M’bokét village (sample ERB-452, Legon Tyo colony), which confirms the presence of the virus in animal reservoirs in the vicinity of the outbreak site. The second result is that the MARV circulating in the Legon Tyo colony is genetically heterogeneous: the second MARV-positive sample (ERB-79) was more similar to the Ozolin group than to Angola-like MARV. Possession of only a short viral sequence from sample ERB-79 (92 bp) did not allow us to build a robust phylogeny (bootstrap values are less than 70%) (Fig 4), but alignment and comparison of nucleotide substitutions showed that this fragment is closest to the isolate PREDICT_SLAB4104 Koebat_SL_2017 (GenBank accession number: MN258362). Whole-genome sequence of this isolate differs from Angola-like MARV by 8.5% and clustered in Ozolin group, which supports the premise that the ERB-79 sample also belongs to the Ozolin group of MARV.

Similar data on genetic diversity were obtained for the MARV found in ERB in Sierra Leone, where two different genetic variants of MARV were found in the ERB colony of Kasewe Cave: Angola-like MARV and MARV phylogenetically close to Ozolin group [2]. Phylogenetically, the sequences of MARV from Guinean ERB and Sierra Leonean ERB are closely related, which indicates that the MARV foci in these countries are connected, and that these ERB colonies are related (common origin, migration, dispersial of individuals).

MARV sequences from ERB of the Legon Tyo colony are not identical to the MARV isolate from the Temessadou M’bokét patient. The MVD patient isolate is close to sequences from the 2005 Angola outbreak with 98% identity and differs by 3.5% from specimen ERB-452 (Legon Tyo ERB colony). A possible explanation for this difference is the small number of sequenced PCR-positive ERB. Another hypothesis is that there are at least two genetic lines within the Angola-like MARV: the first is more adapted to ERB, and the second is adapted to humans.

To summarize, collected evidence is sufficient to recommend actions to prevent possible further MARV spillovers. The exact location of the cave where MARV-positive ERB roost was determined. Furthermore, it is likely that the MARV spillover event can occur not only when visiting the cave but also within the foraging radius of the ERB. We would recommend to local authorities to inform the inhabitants of the surrounding villages that fruit bats in their area are infected with a very dangerous virus; therefore, they should not be caught and eaten. In addition, to prevent possible spillover through contaminated fruit, it is necessary to wash any fruit before eating. We would also recommend avoiding contact with sick and dead monkeys, which can also be a source of MARV.

## Conclusion

In this work, we showed the presence of PCR-positive MARV fruit bats in Guinea. Sequencing linked MARV isolates to the Angola-like lineage. We have found and described two caves in Guéckédou Prefecture where MARV-positive ERB roost. Our findings suggest that MARV is more widely distributed in Guinea than in just a single location in Guéckédou prefecture, and screening of bats in other parts of the country is needed. These results provide the basis for preventive measures against new outbreaks in Guinea.

## Supporting information

S1 Table Georeferenced date on caught animals. (XLSX)

S1 Appendix Phylogenetic trees of four different fragments of Marburg virus. (PDF)
