# Supplementary material for: Marburg virus in Egyptian Rousettus bats in Guinea: Investigation of Marburg virus outbreak origin in 2021
Source: PLoS Negl Trop Dis. 2023 Apr 26;17(4):e0011279. doi: 10.1371/journal.pntd.0011279 (PMC10166547; doi:10.1371/journal.pntd.0011279)
Supplement: S1 Appendix — (PDF) [file pntd.0011279.s002.pdf]

S2 Appendix

Marburg virus in Egyptian Rousettus bats in Guinea: investigation of Marburg virus outbreak origin in 2021

Makenov M.T., Boumbaly S., Tolno R.F., Sacko N., N'FatomaT.L., Mansare O., Kolie B., StukolovaO.A., MorozkinE.S., Kholodilov I.S., Zhurenkova O.B., Fyodorova M.V., Akimkin V.G., Popova A.Yu., Conde N., Boiro M.Y., Karan L.S.

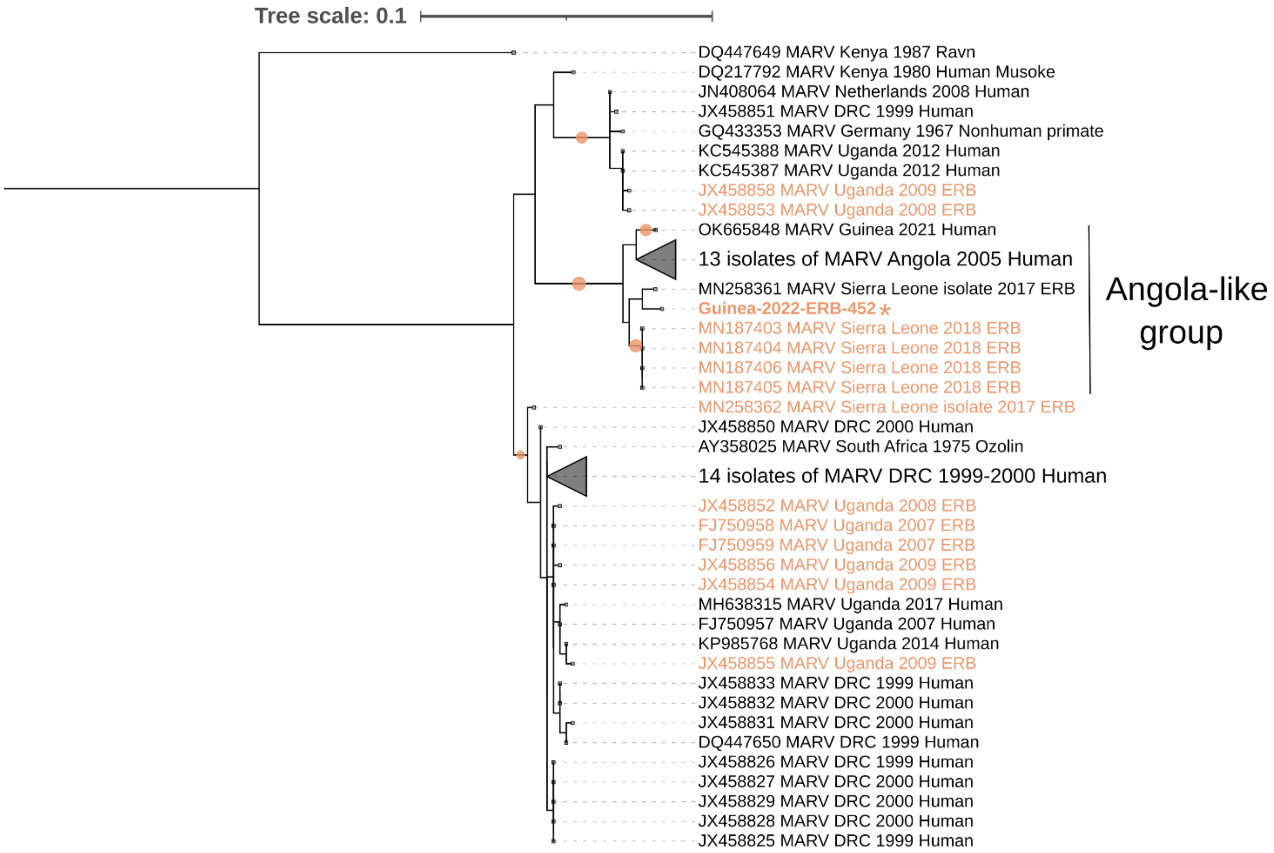

S2 Fig 1. Maximum-likelihood phylogeny of 64 partial Marburg virus nucleoprotein gene (NP gene) fragments with a total length of 459 bp. The tree was constructed using the best-fit nucleotide substitution model (T92+G). Sequences in brown represent those generated from ERB, and sequences in black represent those generated from human and nonhuman primate samples. The sequence from *R. aegyptiacus* ID ERB-452 generated during this study is indicated with an asterisk and bold text. The scale bar indicates the mean number of nucleotide substitutions per site. The filled circles on branches indicate bootstrap values greater than 0.9.

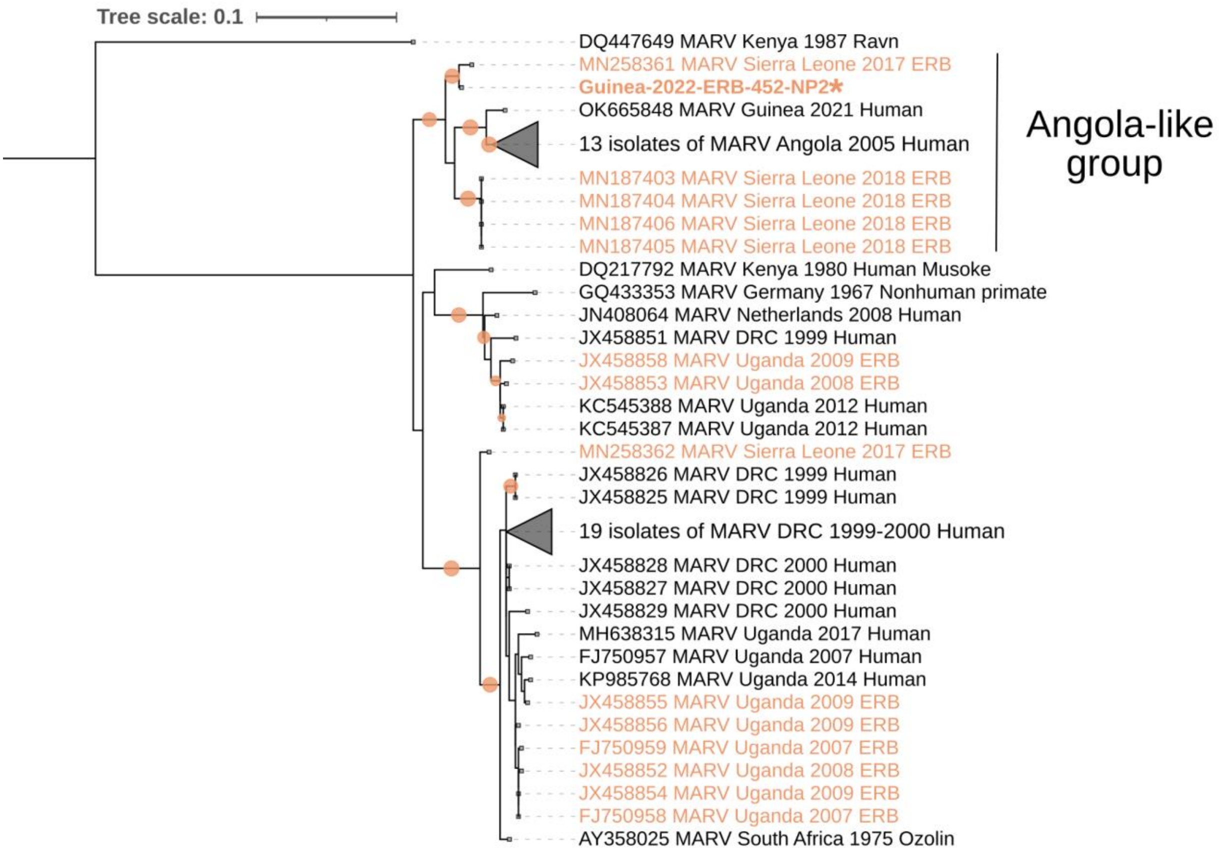

S2 Fig 2. Maximum-likelihood phylogeny of 64 partial Marburg virus nucleoprotein gene (NP gene) fragments with a total length of 457 bp. The tree was constructed using the best-fit nucleotide substitution model (T92+G). Sequences in brown represent those generated from ERB, and sequences in black represent those generated from human and nonhuman primate samples. The sequence from *R. aegyptiacus* ID ERB-452 generated during this study is indicated with an asterisk and bold text. The scale bar indicates the mean number of nucleotide substitutions per site. The filled circles on branches indicate bootstrap values greater than 0.9.

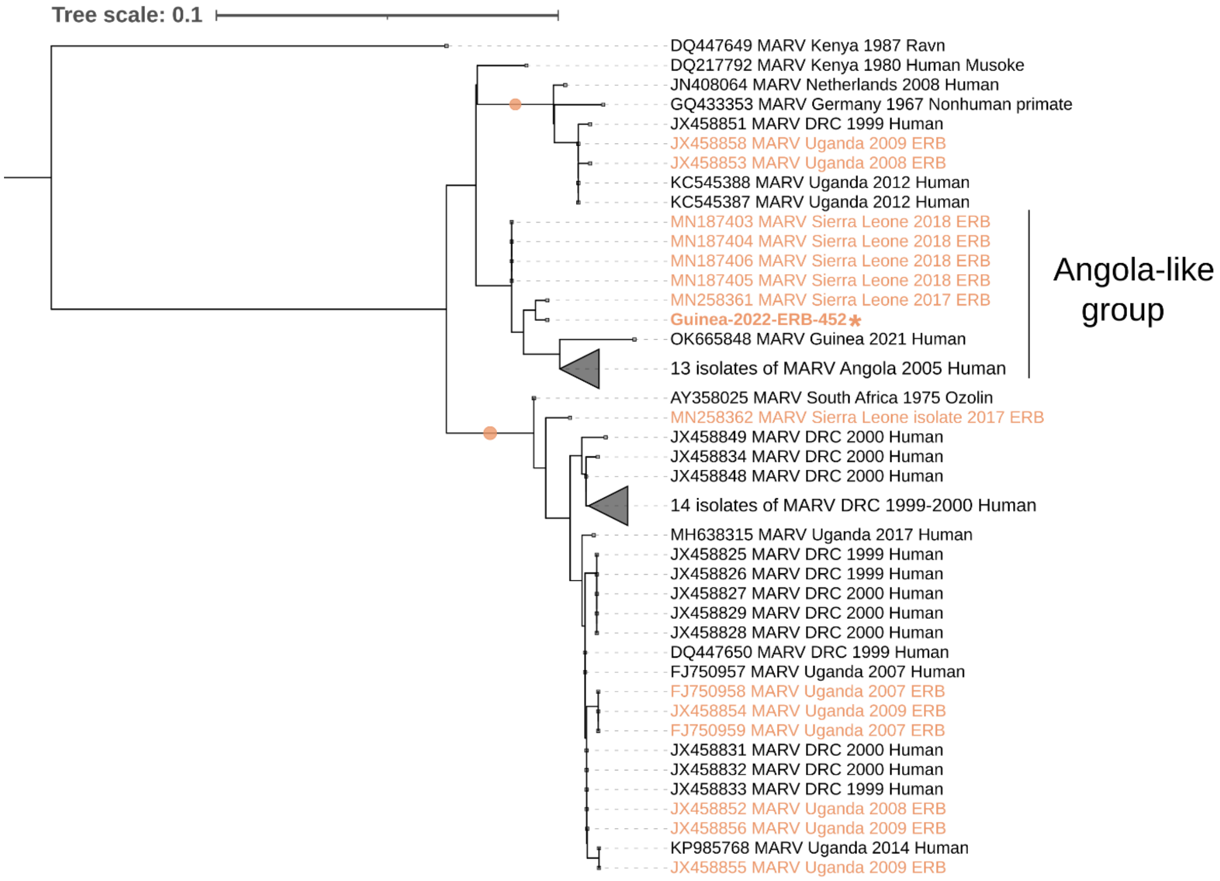

S2 Fig 3. Maximum-likelihood phylogeny of 64 partial Marburg virus VP35 gene fragments with a total length of 300 bp. The tree was constructed using the best-fit nucleotide substitution model (T92+G). Sequences in brown represent those generated from ERB, and sequences in black represent those generated from human and nonhuman primate samples. The sequence from *R. aegyptiacus* ID ERB-452 generated during this study is indicated with an asterisk and bold text. The scale bar indicates the mean number of nucleotide substitutions per site. The filled circles on branches indicate bootstrap values greater than 0.9.

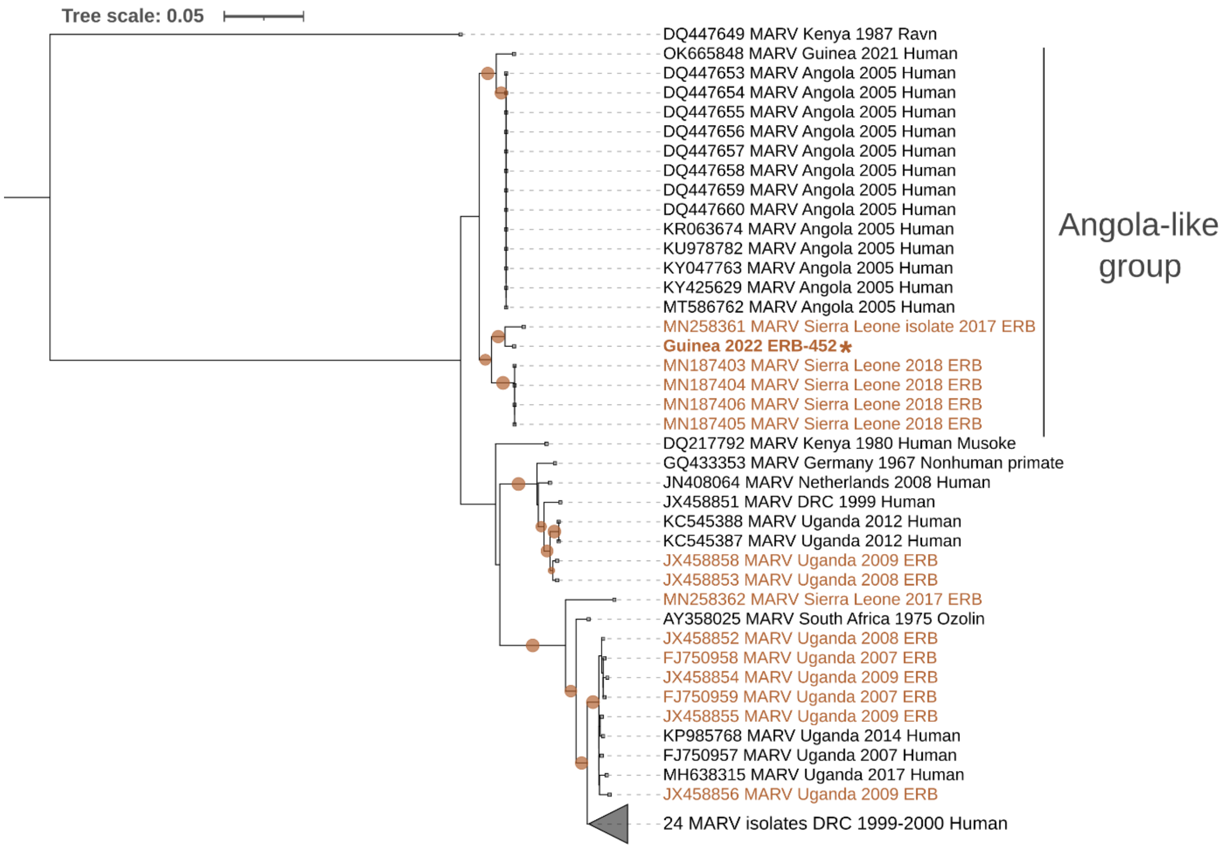

S2 Fig 4. Maximum-likelihood phylogeny of 64 partial Marburg virus VP30 gene fragments with a total length of 1107 bp. The tree was constructed using the best-fit nucleotide substitution model (T92+G). Sequences in brown represent those generated from ERB, and sequences in black represent those generated from human and nonhuman primate samples. The sequence from *R. aegyptiacus* ID ERB-452 generated during this study is indicated with an asterisk and bold text. The scale bar indicates the mean number of nucleotide substitutions per site. The filled circles on branches indicate bootstrap values greater than 0.9.
